# The forest as a classroom: preparing for mental health practice

**DOI:** 10.1186/s12912-016-0128-8

**Published:** 2016-02-01

**Authors:** Marthe Lyngås Eklund, Ireen Ruud, Ellen Karine Grov

**Affiliations:** Institute of Nursing Science, University College of Southeast Norway, PB 7035, 3045 Drammen, Norway; Department of Nursing and Health Promotion, Oslo and Akershus University of Applied Sciences, PB 4 St. Olavs plass, 0130 Oslo, Norway

**Keywords:** Physical activities, Outdoor education, Mental health, Nursing students

## Abstract

**Background:**

Positive effects of physical activity, health promotion and disease prevention, in treatment of mental illnesses are well documented. Mental health practice for nursing students highlights the important connection between physical activities and mental health. This study aims to examine the outcome from nursing students’ participation using *The forest as a classroom.* Students’ collaboration by problem solving, theoretical discussions and performance of activities in the forest serves as a repertoire of non-medical treatment strategies in mental health.

**Methods:**

*The forest as a classroom* was evaluated by means of an ad-hoc questionnaire including both standardized and open-ended questions. Data was analyzed by means of descriptive statistics and content analysis.

**Results:**

The results indicated enhanced knowledge about physical activity and its impact on mental health. However, the nursing students’ experience challenge preserving theoretical exercises outdoor because sensory stimulation took attention away from learning.

**Conclusions:**

For nursing students it is essential to build a repertoire of treatment activities to care for patients having mental health problems. This kind of approach is supported by the students’ learning in the forest. The pilot study highlights the importance of multiple methods of learning in nursing education.

## Background

Several studies focus on nature and the local environment as important factors in health promotion [1–3], for the reduction of anxiety and depression, and for the general improvement of mental health [4–8]. Knowledge of the positive effects of physical activity, health promotion and disease prevention, as well as treatment of physical and mental illnesses, are well documented and therefore recommended to be applied in a variety of community and institutional settings [7, 9]. Physical activity is a non-medical effort to improve mental health, e.g. in order to support housing in the community [10] and coping with daily tasks. Hanson and Jones, 2015, [11] conclude that outdoor walking groups have a wide-range of health benefits, including a reduction of the level of depression. To participants, such walking groups appear to be an acceptable intervention with high levels of adherence and virtually no adverse effects. Another review, from Rosenbaum, Tiedemann, Sherrington, Curtis and Ward, 2014, [12] presented a primary meta-analysis which found that physical activity has a large effect on depressive symptoms, as well as reducing symptoms of schizophrenia and improving anthropometric measures, aerobic capacity and quality of life for people with mental illnesses. An overview of exercises for the prevention and treatment of mental disorders is presented by Dunn and Jewell [13]. The authors refer to this topic as a promising area of research since data indicates that many of these disorders are not treated at all, and there is a significant delay in treatment. Lack of repertoire for non-medical treatment strategies for mental health problems calls attention to preparation for mental health practice during the nursing education. Additionally, the latter mentioned authors underline the importance of more research to integrate mental and physical healthcare for widespread dissemination. Nurses are highly involved in the interdisciplinary team in the community health and in institutions [14, 15]. Our study refers to the context of learning in the forest – a place for knowledge dissemination among nursing students - which might give valuable contribution to the impact of the combination between physical and mental health.

Studying nursing involves the acquisition of knowledge and personal maturation towards a professional role. Practical and theoretical teaching are integrated in the education of nurses in order to make them independent and responsible patient-centered professionals. Approximately 50 % of nursing students at a university college in Norway are in the age group 18-21, and many of them come straight from high school. Classes of about 200 students imply that there is great diversity in the students' previous experience and knowledge. The organization and teaching methods are important motivational factors for learning. Therefore, the teachers use different learning approaches supported by educational theory and methods in teaching/tutoring [16, 17].

### Socio-cultural learning

Cooperation and collaboration are regarded as fundamental to the learning process within the socio-cultural learning perspective [16]. Active participation in social practices is central to the learning process, and communication skills are important tools for learning and performance of the discipline [18]. According to Vygotsky (1934/2001), learning occurs in a social context – first, at an internal psychological level, and further, at an intra-psychological level [19].

Interpretation of this theory might present participation in a social interaction first, while knowledge and integration of skills will eventually be adapted as an individual internal expertise. Learning occurs in interaction with others by exchanging ideas, thoughts and knowledge. Transferred to a teaching situation, teachers and fellow students collaborate by sharing knowledge, experiences, skills and attitudes, which are further converted to their own knowledge by individual integration [19]. The students are expanding their understanding and awareness of a topic internalized in the individual expertise - an expertise that is relevant in clinical situations. By using a method in which students work together, they build relationships, develop social skills and learn to compromise to reach common goals [16, 20]. The social skills are necessary in terms of clinical situations for building relationships, respectful encounters between people, willingness to cooperate and the ability to resolve conflicts. Social skills can be developed in different ways. However, common to the descriptions of these approaches are sharing a community of practice. Vygotsky's description of creative collaboration is provided in a cultural context, or a social community that leads to professional socialization [16]. Wenger (1998) describes the arena of learning as “communities of practice”, and argues that learning occurs when groups of people learn from each other in a social community [21]. Learning in a community implies performing learning activities with aspects of public instruction and guidance. Interaction and collaboration with people are seen as factors for the development of reflection, critical thinking and formation of the students. Learning is promoted through students' active participation in various learning activities. Students ask questions and are in dialogue with fellow students, teachers, texts and practice fields.

### The forest as a learning arena - an outdoor classroom

The students’ motivation, personal interest and previous experience are all essential to learning outcomes [16, 18]. Making learning pleasurable increases motivation and effort, which improves learning outcomes. Sensations through outdoor experiences can affect learning, stimulation and motivation [22]. Nature and other local outdoor venues are “opportunity spaces” for impulses, experiences, physical actions, activities and learning by experience and discovery [16]. Vygotsky's teaching philosophy is based on four concepts: interaction, collaboration tools, characters, and space and creativity [16]. This can be understood as a creative environment with various tools, interpreted as nature’s opportunities. Vygotsky argues that impressions and impulses from the outdoors emerge as a basis for individual experiences followed by reflection and the extension of abstract understanding. However, learning requires teachers’ recognition of students’ pre-understanding and local affiliation.

Szczepanski and Dahlgren (2001) and Szczepanski, Malmer and Dahlgren (2006) add significant focus on students' specific or "earthy" meeting places in the community [23, 24]. “Outdoor education” is suitable for learning when there is desired interplay between experience and reflection based on specific experiences in authentic situations. "Outdoor education" represents an interdisciplinary research and education area that emphasizes the following areas: 1) the learning room moved into the social, natural and cultural landscape, 2) the interaction between sensory-based experiences and theoretical training, and 3) the selected site importance of learning [23, 24].

The forest as a learning arena can help nursing students gain experience from the implementation of learning activities taking place in the forest. It strengthens the students' knowledge of the relationships between outdoor physical activity and mental health in order to prepare for mental health practice. This progression might apply to the students’ practice repertoire in terms of learning and motivation to strengthen management strategies, in this case, related to physical activity in mental health care.

The forest as a learning arena might enhance nursing students’ knowledge of nurses' responsibilities as those initiating physical activities as treatment and rehabilitation strategies in mental health nursing. In addition, common activities in the forest contribute to the process where students and teachers get to know each other and establish confidence prior to clinical practice. Teachers supervising mental health nursing practice experience that students feel insecure when meeting people having mental health problems. Several times the teachers have to deal with students asking questions, such as: "What am I to say? What am I to do?” The forest as a learning arena offers students the necessary experience and knowledge of physical activity and sensory stimulation transferable to mental health practice. Furthermore, the arena prepares students to get started with targeted exercises like walking, jogging, skiing, cycling and fishing. This first step allows for meaningful conversations, interactions and relationship building among the students. Applying knowledge and skills in interaction with others serves as a practice repertoire appropriate in the treatment of people with mental health problems.

Physical inactivity is a major health problem among patients with mental health problems. We have not been able to find any publications on the relationship between learning, motivation and experience of the forest as an outdoor learning arena preparing for mental health practice. We therefore present findings from a pilot study on physical activity competence in the treatment of people with mental health problems. The article describes a new teaching and learning environment to increase knowledge and awareness of using *the forest as a classroom* where the case is physical activities for people with mental health problems.

### Research question

1. To what extent do nursing students describe the impact of the forest as an arena for learning, motivation, understanding of physical activity, and collaboration regarding treatment strategies for people with mental health problems?
2. To what extent do nursing students face the forest as a learning arena when preparing for mental health practice?
3. Is there a difference between the scoring structure among young and old nursing students in the questionnaire developed for an evaluation of this pilot study?
4. How is the factor structure revealed for the participants responding to the questionnaire?
5. How do possible factors correspond to the expected dimensions given from the theory on which the questionnaire is based?

## Methods

### Participants and setting

Lessons in the forest were conducted on the first day of a three-day program preparing for mental health practice. Three university-college professors contributed to the planning and performance of the woodland walk (hereafter referred to as “the hike”). During the hike into the forest, students were divided into groups of about five students, grouped by practice location/practice area. Information and academic content were posted on the university-college's web-based learning platform. Information about the hike's duration, location, clothing, activities, preparing food on a fire, need for stationery and curriculum to prepare for the day were presented electronically on the web. The hike was carried out regardless of the weather conditions. In case of bad weather, there was access to a forest cabin for theoretical work. Table 1 shows that the weather was quite similar despite doing the hike during different times of the year.

**Table 1:** Description of outdoor frames for hike # 1, 2 and 3

Hike	Time	Number of students (n)	Weather conditions	Access to indoor location
1	October 2012	47	+ 8 ^0^C, Rain, windy.	Access to sit under a roof for performing professional work and eating. Preparing food on a fire.
2	January 2013	23	- 10 ^0^C, Snow, windy.	Access to a cabin for performing professional work and eating. Preparing food on a fire.
3	March 2013	34	- 7 ^0^C, Snow.	Access to a cabin for performing professional work and eating. Preparing food on a fire.

### Instrument description

In this study, a questionnaire was used to evaluate teaching and learning outcomes. No standardized psychometric-tested instruments for the purpose of this study were found. To evaluate the educational program and the nursing students’ experience, a structured questionnaire with 13 closed and three open-ended questions were developed. The questionnaire included demographic items about gender and age. The instrument was developed from the theoretical factors of learning and motivation [16, 19], understanding of physical activity [7, 9], and collaboration [19], elaborated on earlier in this article. The survey was designed to evaluate the learning outcomes of *the forest as a learning arena*. Simple psychometric testing of the questionnaire will confirm or deny whether the four dimensions (teaching, motivation, understanding of physical activity and cooperation) appear as the expected pattern. This is a first step in the validation process of a proprietary instrument addressing outdoor learning outcomes.

We wanted to investigate whether there were differences between young and old nursing students' responses to the questionnaire, since this may affect the organization of activities and tasks in the teaching and learning context. The first 12 items in the survey were scored on a 5 point scale (a Likert scale) with the following rated values: 1 = very large extent, 2 = large extent, 3 = moderate extent, 4 = little extent, and 5 = seldom considered, all given as continuous scoring options. Question # 13 was given the options ‘A-F’. The three open questions asked for were regarding the following: 1) a description of the experience of the day, 2) what was particularly good and 3) advice on what could be done differently. The advantage of using open questions is that participants are given the opportunity to provide information and supplementary information regarding matters that are not included in the questionnaire’s other items. In this way, the open questions complement the instrument. The questionnaire was delivered in paper form and submitted anonymously at the end of the day of the hike. Participation was voluntary and submission of a completed questionnaire was considered as consent.

### Analyses

Content analysis was conducted for the text data (the three open questions) [25]. The analysis first involves a simple reading, giving the reader an overall impression of the data. Further, the reading process highlights meaningful units, and the preceding paragraph forms the basis for the selection of sub-themes and themes [25]. Quantitative data were analyzed using SPSS version 20, with descriptive statistics of frequencies, correlation analysis, a t-test for comparison between groups and factor analysis to figure out the grouping of factors and to what degree this structure corresponds to the theoretical dimensions presented. The level of significance was set to 95 %, and all analyses are two-sided.

### Research ethics considerations

An anonymous evaluation survey was performed. No lists of names were used, and, therefore, no permission was required regarding data privacy. Since this study did not aim to collect data on the participants’ health conditions, ethical approval was not necessary, according to Norwegian legislation on health and ethics. The data was treated confidentially and in accordance with the university college’s policy. Participation was voluntary, and the submission of a questionnaire that was filled in, was regarded as consent. To avoid influence from the teachers, the students completed the questionnaire after the teachers had left. An external researcher (the last author listed) has contributed to the analyses of the results and writing of the article.

## Results

The survey was conducted during the autumn of 2011 and spring of 2012 with students in the second year of their Bachelor's degree program in nursing. Out of a total of 133 students in the class, 104 students completed “*The forest as a learning arena”*. All participants completed the questionnaire. Eighty-seven students responded to the question regarding gender, which showed that 6 % are males. This number corresponds to the gender distribution in Norwegian nursing education. Out of the 93 students who stated their age, 75 % were between ages 19 and 29.

Through the analysis of text data from the open-ended questions, two themed areas were discovered: *The forest as a learning arena* and *The forest as a venue for preparation for clinical practice*. Sub-themes and meaningful units to each of these themes are presented in Table 2.

**Table 2:** An overview of the themes, sub-themes and meaningful units

Meaningful units	Sub-themes	Theme
Cold, wet and social. Refreshing. Learning during the activities. A meeting place for relationship building.	Sensual learning. Stimulating learning.	The forest as a learning arena.
Stimulus package for physical activity. Familiarity, friendship and trust.	Action repertoire in mental health. Confidence between students and teachers.	The forest as a venue for preparation for clinical practice.

### The forest as a learning arena

The four meaningful units that emerged from the analysis can be grouped into the sub-themes “*Sensual learning*” and “*Stimulating learning”*. The four connected meaningful units summarize several quotes, which are presented below. "*Cold, wet and social*" is the common denominator of the following statements: "I was cold in the beginning, and I was a little inclined to ramble. I noticed that learning was easier when there was activity involved. I was left with a good experience”, "Getting to know people, socializing”, "Enjoyed working in groups" and "As a group we were demotivated, rather than motivated, by standing still and solving puzzles, and reading attachments and articles in the cold weather.” In the latter quotations, the strong sensory impact of being outdoors and especially the cold weather emerged.

“*Refreshing”* is a term that summarizes the following descriptions: "At first, I had little motivation, but after the hike I felt very good." "The day was somewhat unusual for me, did not want to be there, but was pleasantly surprised", "A chance to learn" and "A delightful day of instruction." “*Learning during the activities”* is shown through the following statements: "Nice to get out and do something else than just listening to a lecture", "Using an outdoor area for learning is great!", "Interesting", "Informative" and "Great opportunity to learn." Descriptions such as "Need more information about the hike ahead", "Less reading material on group assignments during the hike", "Freezing hands when it's cold outside and the fact that it's hard to write in the cold" and "Too cold to solve puzzles outdoors" may indicate that some students need more detailed information about the learning arena before the hike in order to optimize clothing for the weather conditions.

*“A meeting place for relationship building”* describes how students felt about the experiential learning context, which is illustrated by the following quotes: "Learned a lot from discussions with fellow students", "Good to get to know fellow students whom I will join in my clinical practice later on" and "Good discussions along the hike". Through quotes and meaningful units, we highlight learning outcomes and experiences achieved by this teaching approach – an approach that has a strong influence on sensory stimulation.

### The forest as a venue for preparation for practice

This theme consists of two meaningful units and two sub-themes. The sub-themes that emerged were *“Action Repertoire in mental health”* and *“Confidence between students and teachers”.* These two sub-themes characterize the outcome from the setting – the venue. "Stimulus package for physical activity" is a meaningful unit that is supported by the following quotations: "A very good professional reminder and motivation boost!”. ”Bad weather, but great for learning when it comes to physical activity", "Cold, too little physical activity - important to be outside in bad weather". These statements show the importance of educational variation and adaptation to promote physical activity, and indicate that the topic is suitable for outdoor education. The academic motivation, described as "boost" in the citation above, can also be enhanced by the perceptual motivation of being outdoors, even in bad weather. New knowledge about physical activity in mental health was also highlighted in the following quotes: "Informative, useful today in relation to practice", "Increased motivation for physical activity measures in psychiatry", "Have received lots of input for physical activity in psychiatry" and "Was not aware of the importance of physical activity in mental health care".

"Familiarity, friendship and trust" appears in the content analysis and becomes apparent through the following quotations: "Educational scientific questions", "Enjoyed the joint review of the issues", "Important to get to know other students who are in the same practice", "Repetition and good preparation for practice", "Good teacher, got to know her/him in a different, positive way" and "Good to get to know each other before practice".

**Fig. 1:**
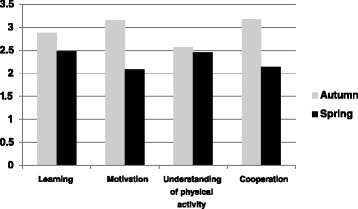
A comparison of mean values for the dimensions; learning, motivation, understanding of physical activity and cooperation, autumn group (*n* = 47) and spring group (*n* = 57)

The students’ overall impression and quality of the hike were evaluated. The students were asked to leave a mark (A-F) indicating this, and according to the answers to the questionnaire, the students experienced the forest as a valuable learning arena. A total of 86 students gave the hike grades A-C, and no evaluations were given the mark “F”. We interpret this as the students having enhanced their preparation for mental health practice, and having gained a broader perspective on the importance of physical activity in mental health nursing as a treatment approach. They also emphasize the importance of getting to know fellow students and teachers before mental health practice. An overall impression of the self-reported evaluation of the dimensions highlighted in the questionnaire is provided in Fig. 1 for the autumn and spring groups respectively.

During the analysis of the questionnaire we examined whether there could be a difference in physical activity between the youngest and oldest students, since experience and habits may affect this. It was assumed that the older students had more experience with physical activity in the forest. In Table 2 we show the average scores for two different groups (median value as the cut-off): young (<29 years) and old (≥29 years) students. There appears to be significant differences between young and old students in three of the questions (#2: Utility of questions and literature, #3: Utility of conversations about tasks, and #8: Working together to solve puzzles), where the old students have higher average scores compared to the young students (Table 3).

**Table 3:** Mean item scores for young and old students respectively

Items	Mean value and (SD) for young students (19–29 years), *n* = 78	Mean value and (SD) for old students (≥29 years), *n* = 15	*p*-value
1. Utility of the forest as a learning arena	2.65 (0.84)	3.00 (0.76)	0.139
2. Utility of questions and literature	2.67 (0.77)	3.13 (0.74)	0.033
3. Utility of conversations about tasks	2.65 (0.77)	3.13 (0.83)	0.032
4. The student’s motivation	2.45 (1.23)	2.40 (1.30)	0.890
5. Changed understanding of physical activity in mental health	2.56 (0.98)	2.67 (1.35)	0.715
6. Utility of preparation before practice	2.90 (0.89)	2.73 (0.88)	0.515
7. Changed motivation for activity	2.58 (0.96)	2.67 (1.18)	0.750
8. Working together to solve puzzles	2.45 (0.96)	3.07 (1.16)	0.030
9. Utility of fellow input	2.67 (0.96)	3.00 (1.0)4	0.241
10. Expectations were met	2.53 (0.88)	2.93 (0.70)	0.101
11. Enjoy being outdoors	2.42 (1.13)	2.67 (1.34)	0.462
12. Hiking in the forest as an appropriate activity	2.49 (1.04)	3.00 (1.00)	0.082

Based on the review of the research literature on physical activity in mental health nursing and theories of learning, the questionnaire was developed to cover the dimensions *learning, motivation, understanding of physical activity* and *cooperation*. Factor analysis revealed a three-dimensional structure when Eigen Value = 1 was used. Questions 1, 2, 3, 6, 7, 8, 10 and 12 were regarding the dimension of learning, questions 4 and 11 were regarding the dimension of motivation, while question 5 represented the understanding of physical activity. The three-dimensional solution gives explained variance R^2^ = 66.7. The dimension *learning* gives an explained variance of 42.1 %. If we use a forced four-part division, the model reveals an explained variance of 74.9 %. The results are displayed in Table 4.

**Table 4:** Factor structure of the four theoretical dimensions of the questionnaire (*n* = 104)

Questions and dimensions	Learning	Motivation	Under- standing of physical activity	Cooperation
*Factor 1. Learning*				
1. Utility of the forest as a learning arena	*0.75*	*-0.17*	*-0.48*	*0.13*
2. Utility of questions and literature	*0.70*	*-0.11*	*-0.44*	*0.07*
3. Utility of conversations about tasks	*0.81*	*-0.03*	*-0.15*	*-0.13*
6. Utility of preparation before practice	*0.67*	*-0.32*	*0.35*	*-0.04*
7. Changed motivation for activity	*0.68*	*-0.29*	*-0.25*	*0.88*
10. Expectations were met	*0.77*	*-0.29*	*-0.25*	*0.16*
12. Hiking in the forest as an appropriate activity	*0.63*	*-0.08*	*0.19*	*0.35*
*Factor 2. Motivation*				
*4.* The student’s motivation	0.47	0.70	0.29	0.27
*11.* Enjoy being outdoors	0.48	0.72	0.07	0.35
*Factor 3. Understanding of physical activity*				
5. Changed understanding of physical activity in mental health	0.47	*-*0.42	0.62	*-*0.11
*Factor 4. Cooperation*				
8. Working together to solve puzzles	0.62	0.37	*-*0.10	*-*0.53
9. Utility of fellow input	0.63	0.33	0.10	-0.54

The high explained variance, which is obtained by correlation analysis, indicates significant overlap between the content of the questions (see Table 5). The only dimension that does not correlate with the other three is *understanding of physical activity*. Hence, this dimension can be interpreted as contextually different from the other dimensions. Cronbach's α = 0.86 suggests highly satisfactory reliability.

**Table 5:** Correlation matrix (Pearson’s *r*) for the questionnaire’s four theoretical dimensions (*n* = 104)

Dimensions	Learning	Motivation	Understanding of physical activity	Cooperation
Learning	-	0.57*	0.34*	0.45*
Motivation	0.57*	-	0.34*	0.51*
Understanding of physical activity	0.34*	0.34*	-	0.19
Cooperation	0.45*	0.51*	0.19	-

*p < 0,01

**Fig. 2:**
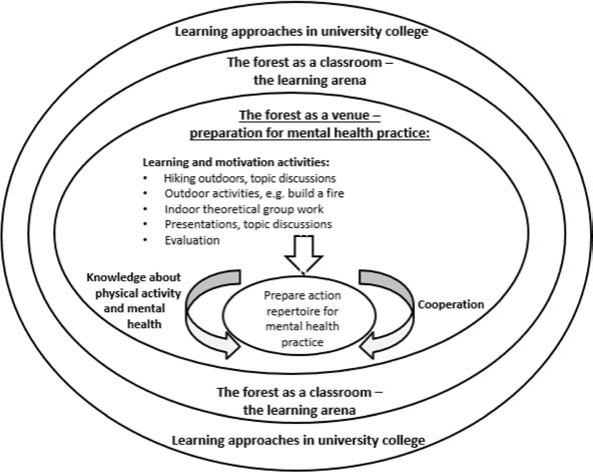
Model: *The forest as a classroom* - preparing for mental health practice

In this pilot study, we show a method of teaching in nursing education on a university college level that requires a theoretical and practical approach. Figure 2 presents thematic areas arising from the qualitative analysis: *The forest as a learning arena* and *The forest as a venue – preparation for mental health practice*.

## Discussion

The main findings from this pilot study show that using *The forest as a classroom* right before mental health practice is important for learning, motivation, understanding of physical activity and collaboration in mental health treatment and promotion. This teaching program may be appropriate for learning outcomes in higher education, in line with the description of Vygotsky (1934/2001) and Lake (2012) [16, 19].

Results showed a difference between young and old students, where the oldest students had higher mean scores, indicating greater experience from learning and collaboration. Since no other significant differences were found in this particular analysis, it is suggested that the composition of groups is not needed in order to differentiate on the basis of age.

The psychometric testing of the questionnaire showed a three-dimensional solution, and a fixed four sharing resulted in a 74.9 % explained variance. There is a high correlation between the three dimensions of learning, motivation and cooperation, however not between these and understanding physical activity. This may indicate that the latter dimension is conceptually different compared to the three pedagogical dimensions [18].

Through the evaluation of the pilot study, students expressed that more time should be spent in the outdoor classroom and more focus should be dedicated to practical learning. Initiatives to create variety in teaching, adapting activities and specific tasks are examples of improvements to the learning activities. The students sense, experience, discover and learn something new in this particular learning context. It increases motivation and thus creates better conditions for learning [16, 22]. Text data from this study shows that sensory input and stimulation promote learning in this arena.

### Learning through activity

From the qualitative analysis two themes emerged: *The forest as a learning arena* and *The forest as a venue - preparation for mental health practice*. Our main impression is that sensory stimulations had a strong impact on the students, and therefore seem to enhance actual learning. As a consequence of this finding, we will increase emphasis on perceptions in a revised version of the questionnaire. Additionally, we will focus on items containing the impact of nature and nature as a classroom. Sensory stimulation, which was emphasized in the feedback from the students, shows the importance of variety and customization of the learning context and educational variety of learning [18, 21, 26]. This particularly applies to the learning community that integrates with the student [19]. Students strengthen their action repertoire of physical activity measures, which might be used in mental health practice, according to national efforts [7, 9]. Since many students are uncertain and insecure and perceive mental health practice as scary, it might be a relief for the students to know each other, the teachers and any relevant treatment strategies. Non-drug approaches in mental health treatment are recommended, and physical activity has shown positive effects for people with mental health problems [3–7, 11–13]. Lack of physical activity is a major health problem among persons with a serious mental illness. Our contribution, however, represents an approach in which nursing students participate to gain knowledge about and experience physical activity in a new learning arena. To perform physical activities space is needed, and Lake (2012) argues for nature and other local outdoor venues as “opportunity spaces” for impulses, experiences, physical actions, activities and learning by experience and discovery [16]. Transferred to a teaching situation, nursing students collaborate and act in the forest – the classroom - by sharing knowledge, experiences, skills and attitudes about physical activity in mental health nursing. This knowledge and experience are further converted to the students’ own action repertoire by individual integration.

Nature is offered to all. Regardless of ethnicity, age and disability, anyone is able to take part in something outdoors. However, there are some principles that can challenge intrinsic motivation, such as too long hikes, unfamiliar environments, and the need for specialized equipment.

It is most probable that students in larger classes will have different experiences and different cultural backgrounds. In this pilot study, some students had no past experience in exploring nature; for some, this hike was their first. Due to lack of information and understanding of the hike, a few students met with inconvenient clothing, no gloves or hat, and/or unsuitable footwear. Therefore, a few students were not able to attend. This suggests that online information is not sufficient for students to understand what the hike entails, for example going through rough terrain in cold weather and participating in activities, such as building a fire and preparing food on a fire. One way of solving this challenge is for teachers to meet the class in advance of the hike and provide the students with detailed information about the nature of the hike, such that the students can acquire the necessary equipment. Furthermore, teachers should encourage students who need support or supplies to take contact.

In addition to learning about physical activity initiatives in mental health work, students will learn about a nurse's responsibility and role as a motivator for the implementation of physical activity [7]. Teachers might, based on feedback from the students, have a clearer focus on the students’ assignments during the hike. Some of the effects of using the forest as a learning arena is that the students pause to examine the trees, pine cones, rocks, streams, views and sky to enhance the experience of being in nature, i.e. experiential learning, as in therapeutic adventure [3]. Most of the students attending Norwegian nursing education are put into large groups for learning and their backgrounds are characterized by diversity [17]. Some students are used to nature and share their experiences, while to others these experiences are new. A hike in the forest is preparation for treatment approaches in mental health practice and experiential environments. Hiking is highlighted as a physical activity providing learning experience from nature and sensory stimulation and promotes collaboration. The student learns together with others to accrue an action repertoire of physical activity, and it is expected that they will later on transfer learning from the social context into their individual competence [19, 20].

The results from the questionnaire show that 86 out of 104 students had a good overall impression of the hike (these students answered in the range A-C to question #13). Analyses show that the group that attended during spring had better scores compared to the autumn group, apart from understanding the dimension of physical activity. The two themes (Table 2) with associated sub-themes that emerged from the content analysis are consistent with the four dimensions of the questionnaire. The focus is on learning, learning context, motivation, sensory stimulation and cooperation for preparation for clinical mental health practice. An action repertoire for the treatment and monitoring of patients, as well as trust between students and teachers, are outcomes of this. Students may apply some of the approaches used during the hike in their mental health practice and be aware of the importance of confidence and a repertoire of a variety of approaches in the treatment of patients.

### Practical arrangements for the arena

It is useful to have access to a heated cabin when it is cold and wet in order to work with theoretical tasks, i.e. writing and applying the available literature. Conversations and discussions using literature strengthen joint activity and cooperation. Joint activities, such as making a fire, preparing food on the fire and having conversations around the fire inspires learning, regardless of the weather. It is important for social communities to provide such opportunities to get acquainted and to learn such activities. However, if weather conditions are poor, students might have a negative experience and report the opposite to what is the purpose of the hike, i.e. they do not want to use nature, either in their own time or as a future arena for patient treatment. Sensations may seem both positive and negative, especially when complete focus is on strong sensations, such as a beautiful landscape view and cold hands and feet, preventing other sensations that stimulate learning to occur.

In nursing education, research on students’ learning and teachers' challenges to promote the best possible learning environment is well documented [18]. The desire to transfer and adapt the knowledge of the specific practical activities can make the subject more interesting and easier to understand. In this learning process, the actual hike is a practical exercise – an experiential ground floor. The hike is an example of an activity that has an impact on the mental and physical health status. The experiential learning described might therefore serve as a way to support and treat people with mental health problems.

### The study's strengths and limitations

The strength of the study is structured implementation of the teaching program focusing on exercises that provide an action repertoire for treatment and follow-up in mental health practice. Specification of Vygotsky's idea of learning in creative collaboration with fellow students and teachers [16] is the inspiration for this pilot study, where the forest serves as the creative collaboration venue - the ability classroom. Whether the patients are dwelling in the community or in an institution, different outdoor venues might be available for physical activities, here exemplified from Norway with the forest as a classroom. Further research is recommended to examine other outdoor venues, e.g. parks, as an arena for physical activity in mental health.

The teaching program evaluated included both qualitative and quantitative data and demonstrated that the questionnaire is suitable for evaluation of this type of activity, although the questionnaire has potential for improvement. As far as we know, the forest as a learning arena is not implemented in graduate education on Bachelor level. However, kinder gardens and elementary schools have used this arena with great success. The study's limitations include the sample consisting of only first year nursing students at one university-college in Norway and the number of participants available during the data collection period. Thus, the sample limits statistical analyses.

Physical activity and preparation practices might have different approaches at other universities, and the findings from this study cannot be generalized to other nursing programs. For students who do not have any experience with the use of nature, the hike yields uncertainty when it comes to coping with related challenges, both psychological in terms of high and slippery mountains and physical, e.g. inappropriate equipment, clothing, shoes etc. Based on the results from this study, a more extensive interview-based study is recommended as a useful additional piece of work. Additionally, as Keniger et al. (2013) [27] argue, research lacks documentation about the mechanisms important for the effects of e.g. psychological well-being; i.e. what triggers a beneficial interaction. Further research on hiking as an intervention for students in their learning process and relationship building is therefore recommended. The transfer value of this study for targeted work with physical activity to other patient groups, such as people with heart disease, obesity and diabetes, is suggested for further research.

## Conclusion

Physical activity, the use of nature, outdoor activities and public health are essential for nurses who work in mental health care. The results from the study regarding learning activities in the forest show the importance of having an action repertoire of activities to meet patients' needs for varied physical activities. This pilot study highlights the importance of multiple methods of learning in nursing education, with variation in the use of learning context and approaches for supporting quality in teaching and learning. From the qualitative analysis of text data, two main themes emerged: *The forest as a learning arena* and *The forest as a venue - preparation for mental health practice*. The four dimensions of the questionnaire, consisting of learning, motivation, understanding of physical activity and collaboration, provide 74.9 % explained variance. It is beneficial to include questions regarding the importance of experience in nature and sensations in a revised version of the questionnaire. We recommend performing more research on using the nature as a classroom for the connection between physical activity and mental health nursing.
